# GcForest-based compound-protein interaction prediction model and its application in discovering small-molecule drugs targeting CD47

**DOI:** 10.3389/fchem.2023.1292869

**Published:** 2023-10-20

**Authors:** Wenying Shan, Lvqi Chen, Hao Xu, Qinghao Zhong, Yinqiu Xu, Hequan Yao, Kejiang Lin, Xuanyi Li

**Affiliations:** ^1^ Department of Medicinal Chemistry, School of Pharmacy, China Pharmaceutical University, Nanjing, China; ^2^ Faculty of Health Sciences, University of Macau, Macau, China; ^3^ Institute of Chemical Industry of Forest Products, Chinese Academy of Forestry, Nanjing, China; ^4^ National Engineering Laboratory for Biomass Chemical Utilization, Nanjing, China; ^5^ School of Humanities and Social Sciences, The Chinese University of Hong Kong, Shenzhen, China; ^6^ Department of Pharmacy, Nanjing Drum Tower Hospital, Affiliated Hospital of Medical School, Nanjing University, Nanjing, China

**Keywords:** artificial intelligence, word2vec, GcForest, compound-protein interaction prediction, small-molecule CD47 inhibitors

## Abstract

Identifying compound–protein interaction plays a vital role in drug discovery. Artificial intelligence (AI), especially machine learning (ML) and deep learning (DL) algorithms, are playing increasingly important roles in compound-protein interaction (CPI) prediction. However, ML relies on learning from large sample data. And the CPI for specific target often has a small amount of data available. To overcome the dilemma, we propose a virtual screening model, in which word2vec is used as an embedding tool to generate low-dimensional vectors of SMILES of compounds and amino acid sequences of proteins, and the modified multi-grained cascade forest based gcForest is used as the classifier. This proposed method is capable of constructing a model from raw data, adjusting model complexity according to the scale of datasets, especially for small scale datasets, and is robust with few hyper-parameters and without over-fitting. We found that the proposed model is superior to other CPI prediction models and performs well on the constructed challenging dataset. We finally predicted 2 new inhibitors for clusters of differentiation 47(CD47) which has few known inhibitors. The IC_50_s of enzyme activities of these 2 new small molecular inhibitors targeting CD47-SIRPα interaction are 3.57 and 4.79 μM respectively. These results fully demonstrate the competence of this concise but efficient tool for CPI prediction.

## Graphical Abstract

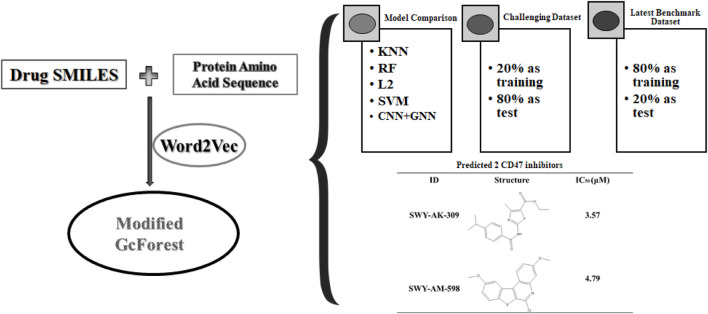

## 1 Introduction

Drug discovery is a time and resource-consuming process. About 2.6 billion US dollars is needed for developing a new drug and 17 years for FDA approval (Mullard, 2014). Accurate prediction of compound–protein interactions (CPI) may help lead identification, which plays a vital role in drug discovery. And ML has quickly penetrated various aspects of drug discovery, including the successful application in CPI prediction, such as the recently proposed CPI model called DeepLPI and CoaDTI (Rifaioglu et al., 2019; Shan et al., 2021; Huang et al., 2022a; Jung et al., 2022; Su et al., 2022; Wei et al., 2022; Wong et al., 2023; Zheng et al., 2023).

However, there are several obstacles that hinder accurate predictions of compound-protein interactions. One of these challenges is the complexity of biological systems. Compound-protein interactions occur within the context of intricate cellular pathways and networks. How to represent these proteins and small molecules for ML is the frist issue we need to face. The extracted chemical and genomic information of compounds and proteins, such as the substructures of compounds, physicochemical and biomedical properties of proteins, were usually considered as input in ML-based methods for CPI prediction (Ma et al., 2019; Sachdev and Gupta, 2019; Tsubaki et al., 2019; Lin et al., 2021; Xu et al., 2019; Jung et al., 2022). Differently, several ML-based models such as DeepCPI, DeepConvolutional DTI, GraphDTI, DeepLPI and CoaDTI enable the process of raw data, in which DeepLPI and CoaDTI are all well-known end-to-end frames using raw data of compounds and proteins, such as SMILES of compounds and amino acid sequence of proteins (Wen et al., 2017; Li et al., 2019a; Ester, 2019; Huang et al., 2022a; Wei et al., 2022). These DL-based models have the defects of many hyper-parameters, which makes the training and theoretical analysis difficult. In addition, DL-based models are often over-fitting and with lower accuracy on small-scale datasets, which are obstacles to CPI predictions (Li et al., 2019b; Lee et al., 2019; Wan et al., 2019; Liu et al., 2021).

GcForest (Zhou and Feng, 2017) is an ensemble decision tree learning algorithm with unique features. GcForest can adaptively determine the model complexity and avoid overfitting, in which 3-fold cross validation is used in the training process, and the training stops when the performance of the model is not significantly improved. GcForest could be trained easily with few hyper-parameters, which enable the robust and excellent performances on both large-scale and small-scale datasets. GcForest does not require fine-tuning of parameters such as learning rate, number of hidden units or depth of layers as in DL. Instead, it only needs to set some basic parameters such as number of trees, number of features and number of classes for each random forest layer (Zhou and Feng, 2017). While task-specific tuning is carried out for DL, gcForest outperformed DL with just the same configuration (Zhou and Feng, 2017). Besides, the training of gcForest is efficient and robust even with low computing power computers.

**FIGURE 1:**
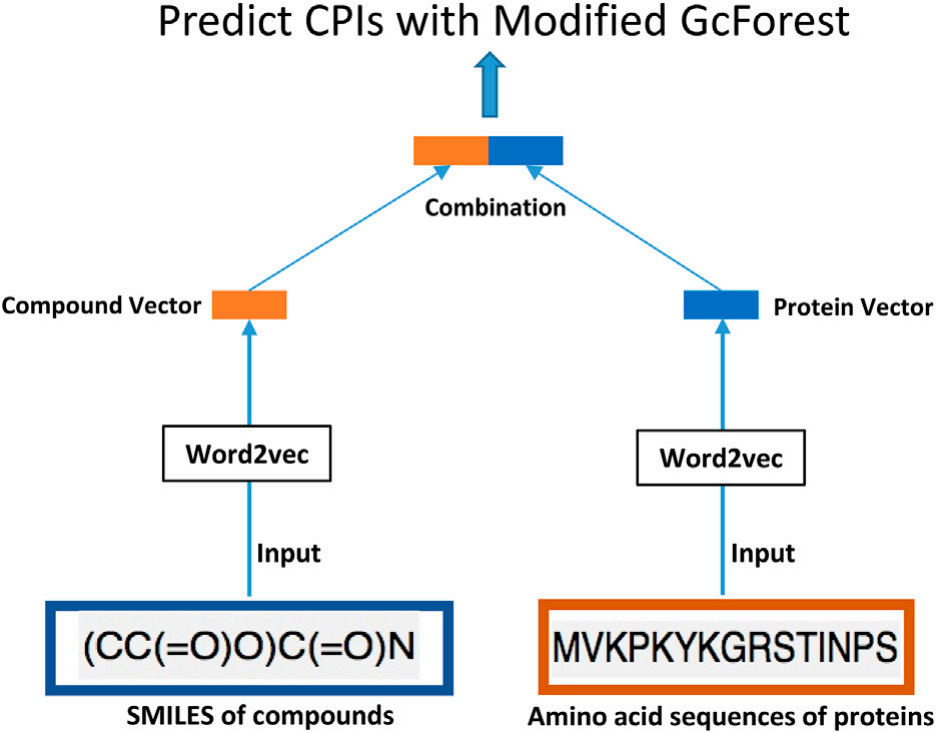
A flow-chart of the data preparation process for our proposed model.

In this article, we innovatively proposed the combination of word2vec (Mikolov et al., 2013) and the modified concise but efficient gcForest (Zhou and Feng, 2017) classifier as a new CPI prediction model. As shown in Figure 1, the transformed embedding vectors of compounds and proteins obtained from word2vec are used as input to the modified gcForest classifier to predict the CPIs. Although the most current CPI prediction models all have excellent performances on the benchmarks, few of them could be taken into realistic application to find new drugs for a specific protein (Lim et al., 2021).

Nowadays, new proposed models have to be proved to be useful through solving experimental problems. In our research, we took the modified gcForest into realistic application to screen the new compounds for an anti-tumor immune target, cluster of differentiation 47 (CD47). CD47 is an immunoglobulin which is overexpressed in many different tumor cells. Its interaction with signal-regulatory protein α (SIRPα) can help cancer cells escape phagocytosis, which is a promising anti-cancer target. Currently only one small molecular inhibitor has entered the phase of clinical development (Burgess et al., 2020; Yu et al., 2021; Qu et al., 2022). As a result, our model predicted 2 compounds that inhibited CD47 and SIRPα interaction with IC50 values of 3.57 and 4.79 μM, respectively. These results fully demonstrate the competence of the proposed CPI prediction model, especially for targets with few known drugs.

## 2 Materials and methods

### 2.1 Construction and validation of the proposed modified GcForest CPI prediction model

#### 2.1.1 Preparation of the benchmark datasets

The performance of gcforest in the face recognition task is better than that of Convolutional Neural Network (CNN), which has more obvious advantages in the case of less training data (Zhou and Feng, 2017). Our research group previously constructed a 2D image recognition model based on CNN, which predicted two active CDK4 inhibitors (Xu et al., 2018), namely, indocyanine green and candesartan, with IC_50_ values of 2.0 and 5.2 μM, respectively, this model used 2D images of structures of drugs as input. In order to evaluate the performance of gcforest with less training data, we compared the performance of gcforest with CNN based CDK4 drug screening model, we used the same dataset of the CNN based CDK4 drug screening model (Xu et al., 2018), which contains a total amount of 1,040 active and inactive 2D images of structures of drugs. It is worth noting that the CNN based CDK4 inhibitor screening model increased the amount of training data by rotating the images of inactive compounds. And we deleted the rotated compound images, remaining a total amount of 777 active and inactive 2D images of structures of drugs. The code of CNN based CDK4 drug screening model include the datasets can be obtained from Github (https://github.com/Xyqii/intuitive-drug).

Most datasets used in the CPI prediction methods contain positive data and randomly generated negative data, while these randomly generated negative data may contain true positive data. Thus, it is vital to construct reliable true negative CPI datasets (Liu et al., 2015). We downloaded the datasets provided by Liu (Liu et al., 2015) who constructed reliable true negative CPI datasets. Liu constructed human and *C. elegans* datasets, which are based on the assumption that the proteins dissimilar to any known/predicted target of a given compound are not much likely to be targeted by the compound and *vice versa* (Liu et al., 2015). Positive samples of the datasets were retrieved from two manually curated databases: DrugBank 4.1 (Wishart et al., 2008) and Matador (Gunther et al., 2008). The Tanimoto coefficient was used to measure the similarity between compounds and proteins, and the negative samples that have a low similarity score with any positive sample were selected (Liu et al., 2015). The ratio of positive and negative samples was 1:1. We deleted duplications and drugs whose length of the SMILES string was less than 3 (to train word2vec). In the end, the human dataset contains 5,995 interactions between 2,724 unique compounds and 2,001 unique proteins; the *C. elegans* dataset contains 6,527 interactions between 1,763 unique compounds and 1869 unique proteins. We used these two datasets, and 80% of each was used as the training set and 20% as the test set.

**FIGURE 2:**
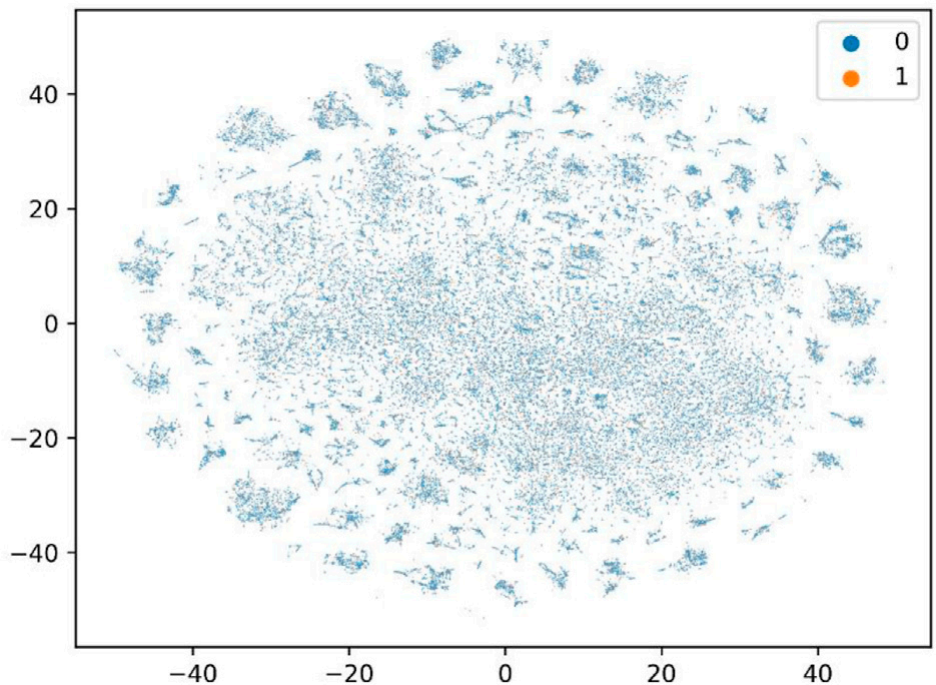
T-SNE to visualize the distribution of the constructed challenging dataset.

To further evaluate the performance of our model, we constructed a large-scale dataset, and we randomly selected 20% as the training set and 80% as the test set, which is more challenging and more in line with the real virtual screening scene where the number of known active molecules towards a specific target is small. We used ChEMBL data retrieved from BindingDB (Liu et al., 2007). BindingDB is a public and available database that contains measured binding affinity data and is focused on CPIs. We deleted duplications and drugs whose length of the SMILES string was less than 3. We then constructed the positive and negative datasets using the standard that the value of IC50 or Ki was less than or equal to 1 µM was filtered as positive data, and the value of IC50 or Ki of that was greater than or equal to 30 µM was filtered as negative data. We set the ratio of the positive data with the negative data as 1:3. Finally, the dataset we constructed had 29,320 positive data and 87,960 negative data, in which 3,420 unique proteins and 80,931 unique small molecules are included. As shown in Figure 2, T-SNE was used to visualize the distribution of the whole challenging dataset, each point represents a pair of compound and protein, the orange points represent the dataset for training, and the blue points represent the dataset for test. We can see that the training and test sets have a broad and similar distribution and that the random splitting is rational.

**FIGURE 3:**
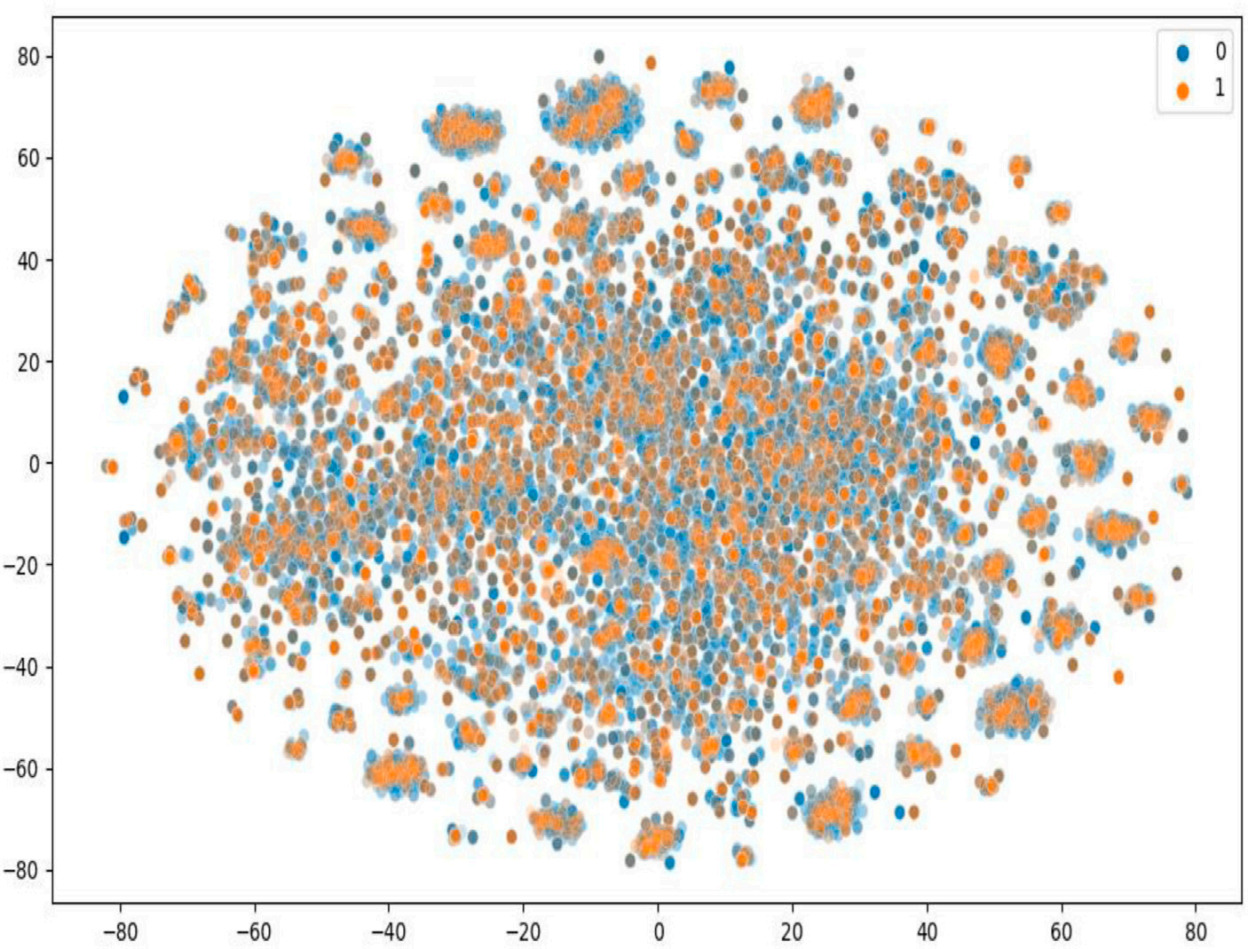
T-SNE to visualize the distribution of the constructed latest benchmark dataset.

Besides the old and classic humans and *C. elegans* datasets, we also made our model compared with other state of art models using the latest benchmark datasets, BindingDB. We downloaded all the CPI data in the dataset version 2022-12-01. The initial dataset has 2,627,702 CPI measurements, 1,129,664 compounds and 8,946 targets. We conducted the following pretreatment to achieve more convincing data. First, only the CPI data has K_d_ value was retained. K_d_ is a direct measure of the binding affinity between a compound and a protein, which reflects the strength of their interaction. Other measures, such as IC_50_, EC_50_, or K_i_, may be affected by various factors, such as the assay conditions, the protocol, the sources of enzymes, the substrate concentration, or the presence of other molecules. Second, the CPI value containing “<” or “>” was deleted. Third, K_d_ values less or equal to 1,000 nM were filtered as positive data, and larger than 1,000 nM as negative data. Our preprocessed dataset has vigorous and convincing standards similar to DeepLPI (Wei et al., 2022). We set the ratio of the positive data with the negative data as 1:4. The processed datasets were randomly shuffled and 80% of which was selected as training data, the remaining 20% as test data. Finally, there are 39,563 pair CPIs containing 15,950 drugs and 1,707 proteins in the whole processed benchmark dataset. In the training dataset, there are 31,648 CPIs containing 13,541 drugs and 1,620 proteins. And in the test dataset, there are 7,915 CPIs containing 4,555 drugs and 1,162 proteins. As shown in Figure 3, T-SNE was used to visualize the distribution of the constructed latest benchmark dataset, each point represent a pair of compound and protein, the blue points represent the dataset for training, and the orange points represent the dataset for test. We can see that the training and test sets have a broad and similar distribution and that the random splitting is rational.

The above benchmark datasets were collected for model construction and validation, construction information of the above datasets is summarized in Table 1.

**TABLE 1:** Construction information of the benchmark datasets.

	Human	*C.elegans*	The constructed challenging dataset	The constructed latest BindingDB dataset
Number of compounds	2,724	1,763	80,931	15,950
Number of proteins	2,001	1,869	3,420	1,707
Number of positive CPIs	2,997	3,263	29,320	26,209
Number of negative CPIs	2,997	3,263	87,960	13,354

#### 2.1.2 Generation of compound-protein feature vectors

As shown in Figure 1, the transformed embedding vectors of compounds and proteins obtained separately from word2vec (Mikolov et al., 2013) are then combined to be used as input to the modified gcForest classifier to predict the CPIs. In particular, we used the skip-gram method of negative sampling to train the word embedding model and learn the context relationship between the words in the sentences. In our study, the dataset used to train word2vec is all the 80,931 pairs of CPI data in the challenging dataset. We followed the method of Wan’s (Wan et al., 2019) to parse SMILES and protein sequences into words of length 3. The SMILES of drugs and amino acid sequence of the targets were regarded as “sentences”, and every three non-overlapping amino acids and SMILES were regarded as a “word” (Wan et al., 2019). We followed the principle of commonly trained word2vec to select the hyper-parameters of skip-gram (Asgari and Mofrad, 2015). More specifically, the size of the context window is set to b = 12, the number of negative samples is set to k = 15, and the embedded dimension is set to d = 32. This dimension is far less than 100, which is the most commonly used embedded dimension in previous research (Wan et al., 2019), thus effectively reducing the dimensions of the input data for the same sample. We have trained word2vec separately on the SMILES of compounds and the amino acid sequences of proteins, and obtained the low-dimensional vectors of them. Then, we have combined the vectors of compounds and proteins to be used as input to the modified gcForest classifier to predict the CPIs. We used a simple merging method to combine the low-dimensional vectors of proteins and compounds obtained separately from word2vec. Specifically, we have concatenated the 32-dimensional vector of the compound and the 32-dimensional vector of the protein, resulting in a 64-dimensional vector that is used as input to the classifier.

One of the advantages of using word2vec is that it is a simple and fast method that can generate features of proteins and ligands from their sequences or SMILES representations, without requiring any additional information or preprocessing. Word2vec can capture the semantic similarity and the local context of the amino acids in the sequences or SMILES representations, and can produce fixed-length vector features that are suitable for downstream machine learning tasks. Moreover, word2vec is a well-established and widely used method that has been proven to be effective in various domains, such as natural language processing, computer vision, and bioinformatics. However, one of the disadvantages of using word2vec is that it may not be able to capture the complex and high-dimensional features of compounds and proteins that are relevant for CPI prediction, such as their 3D structures, physicochemical properties, functional domains, binding sites, interactions, etc., which may affect their binding affinity and specificity, but word2vec just learn the semantic similarity and the local context of raw data of the amino acids in the sequences or SMILES without further process, which make it easily interpretable or explainable. and Yu Fang Zhang (Zhang et al., 2019) has explained the biochemical implications of word2vec generated features, that is the proteins and compounds with the similar sequences which indicate similar biochemical implications are close to each other. Therefore, a possible future direction for improving our method is to use more advanced language models that can learn compound and protein features, such as ESM (Arndt et al., 2023), ProGen (Madani et al., 2020) ChemBERTa-2 (Ahmad et al., 2022) CGR (Huang et al., 2022b) AminoBERT (Chowdhury et al., 2022) and MMseqs2 (Steinegger and Söding, 2017). These language models are based on deep neural networks, such as transformers or recurrent neural networks that can learn rich and contextualized features of compounds and proteins from their sequences or SMILES representations. These language models can also leverage the pre-training and fine-tuning techniques to transfer the knowledge learned from large-scale unlabeled data to specific CPI prediction tasks. Moreover, these language models can handle the large vocabulary size and the sparsity of the data in CPI prediction, and can also adapt to the new or unseen compounds or proteins by using dynamic or self-attention mechanisms. These language models may be able to achieve better performance and robustness than word2vec in CPI prediction. However, using these advanced language models may also have some challenges and drawbacks. For example, these language models may require more computational resources and time to train and evaluate than word2vec. These language models may also suffer from overfitting or underfitting problems due to the complexity of their architectures or the heterogeneity of their data sources. Moreover, these language models may not be easily interpretable or explainable, which may hinder their practical use in drug discovery.

#### 2.1.3 Architecture and training process of the modified GcForest

GcForest is robust to hyper-parameter adjustments. Even when working with diverse data from various domains, it can achieve outstanding performance with the same default setting. The 3-fold cross validation is employed to make it reliable and consistent across various data splits without adjusting the random seeds. And gcForest specifically divides the training set into two components, the growing set and the estimating set. The growing set is used to grow the cascade and the estimating set to estimate performance. The cascade’s development stops and the number of levels is acquired if adding a new level does not significantly increase the performance. And gcForest uses 20% of the training data for estimating set and 80% for growing set (Zhou and Feng, 2017).

There are two stages in the training process of multi-grained cascade forest model: Multi-grained scanning and cascade forest. The multi-grained scanning was used to extract feature vectors through different sliding windows, and the cascade forest was applied to obtain the prediction results through multiple cascades forest. And the following features enable gcForest to avoid overfitting: gcForest uses Multi-Grained Scanning to split data, which can increase the diversity and randomness of data, and the Cascade Structure is used to increase the complexity of the model layer by layer, and performs cross validation at each layer to decide whether to continue adding layers. GcForest uses Random Forest as a basic classifier, generating multiple random forests at each layer and combining their results, which can improve the robustness and accuracy of the model (Zhou and Feng, 2017).

**FIGURE 4:**
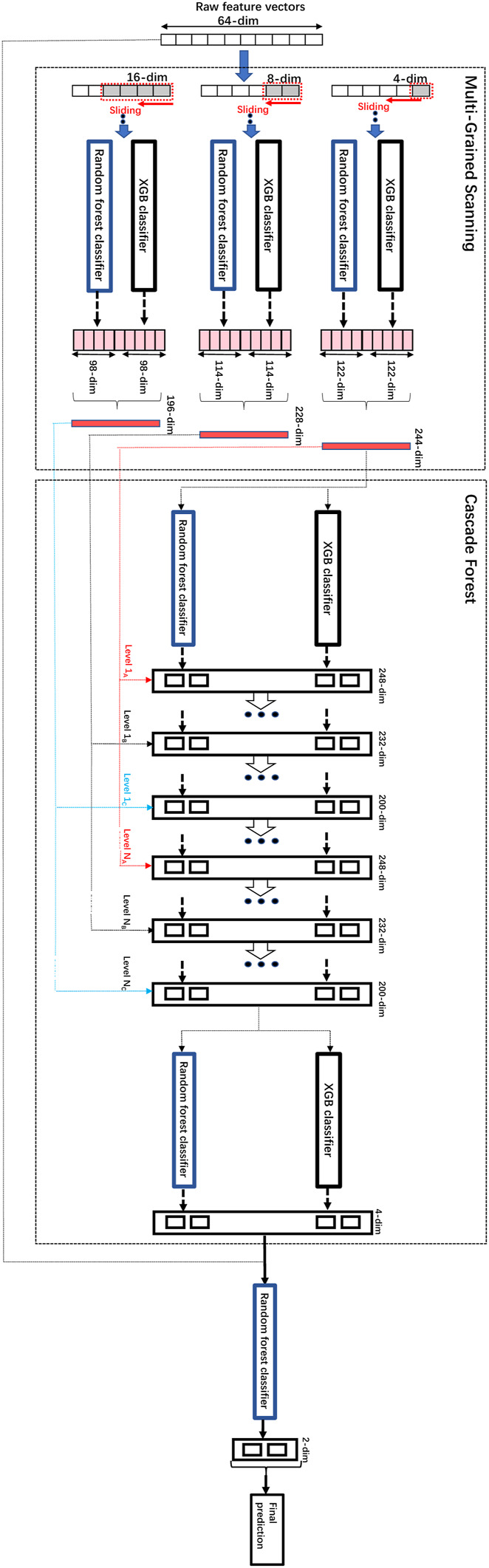
The architecture of the modified gcForest and its training process.

We modified the parameters and the architecture of gcForest (Zhou and Feng, 2017). The modifications include the parameters to adapt to the input of low-dimensional embedding vectors generated by word2vec, specifically, the dimensions of the raw data, the dimensions of the sliding windows, the categories of the classifiers used inside as well as the added final Random Forest classifier layer to improve performance on top of the initial gcForest. The original input dimension of the combined feature vectors obtained by word2vec is 64, and 3 different sizes of sliding windows are used for multi-grained scanning, 4, 8 and 16 respectively. The multi-grand scanning and the cascade forest components both utilized two kinds of classifiers, XGB classifier and random forest classifier, respectively. In order to further improve the performance, we added a random forest classifier on top of the above architecture, and the above transformed data combined with the original vectors obtained by word2vec were used to train the final random forest classifier on top to obtain the final predictions of CPIs. The architecture of the modified gcForest and its training process are shown in Figure 4.

#### 2.1.4 Metrics for model evaluation

We used accuracy (ACC), precision, AUC (area under the ROC curve), sensitivity (SE) and specificity (SP or recall) to evaluate and compare the performance of our model with other CPI models. The area under the receiver operating curve (AUC) is calculated by plotting the true positive rate versus the false positive rate for varying decision thresholds. The closer the value of AUC to 1, indicating the better performance of the model. The metrics above are calculated using the formulas as follows:

$$Accuracy=\frac{TP+TN}{TP+FP+TN+FN}$$

$$Sensitivity=\frac{TP}{TP+FN}$$

$$Specificity=\frac{TN}{TN+FP}$$

$$Precision=\frac{TP}{\left(TP+FP\right)}$$

TP: number of true positives, FN: number of false negatives, TN: number of true negatives, FP: number of false positives.

### 2.2 Application to screen new CD47 inhibitors

#### 2.2.1 Preparation of datasets of known CD47 small molecular inhibitors

We retrieved 68 CD47 small molecule inhibitors from Reaxys (Levy, 2014), Cortellis (Mulvihill, 2012) and BindingDB (Liu et al., 2007), among which, 2 CD47 small molecule inhibitors have activities (IC_50_ or binding affinity) of less than 1 µM. The activities and structures of the 2 known most active small molecule inhibitors are shown in Table 2.

**TABLE 2:** Information of the 2 known most active CD47 small molecular inhibitors.

Structure	Activity (nM)	Source
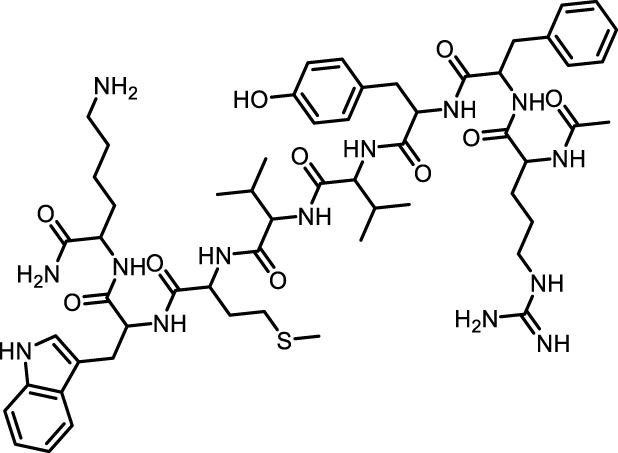	771	BindingDB ID: CHEMBL3946082
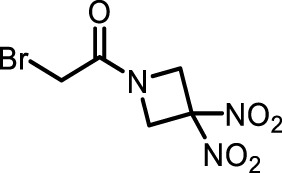	62.5	Cortellis ID: 725,899

#### 2.2.2 Preparation of the commercial library for virtual screening

We downloaded the available small-molecule compounds lists from Specs, a commercial library (https://www.specs.net/), and after preprocessing, 309,246 small molecules were obtained for virtual screening.

#### 2.2.3 Models to screen new CD47 small molecular inhibitors, visualization of compound-protein interactions and visualization of the similarities between the screened inhibitors and the known inhibitors

We trained the proposed models with 2 different datasets to screen the commercial library individually. The first model was trained with the entire challenging dataset, and the second model was trained with the entire datasets of the 68 known CD47 small molecular inhibitors. The second model was only trained with the known CD47 small molecular inhibitors without the CD47 protein information, since the protein sequences are the same in the process of training and screening new CD47 inhibitors. Therefore, we think that the protein information is redundant and irrelevant for the second model, which is only trained with the known CD47 small molecular inhibitors. The model trained on the large scale dataset, namely, the challenging dataset, which contains a huge number of different compound-protein interactions, can capture the general features and patterns of molecular recognition and binding. This model can provide a broad and unbiased screening of potential inhibitors for our target protein. The second model aims to learn the specific characteristics and preferences of the ligands that can bind to CD47, rather than the general features and patterns of molecular recognition and binding. By using both models, we can combine the advantages of each model and obtain a more comprehensive and reliable screening result.

The 3D structure of the CD47 protein was obtained from the RCSB database, ID: 2JJS. At present, there is no available crystal structure of CD47 with its active small molecule ligand. We used Discovery Studio 2016 to dock the known inhibitors and the predicted inhibitors for CD47 and visualize the compound-protein interactions.

We used Find Similar Molecules by Numeric Properties function in Discovery studio 2016 to visualize the similarities between the screened two active CD47 inhibitors with the 68 known inhibitors. The Find Similar Molecules by Numeric Properties protocol finds ligands that have similar properties compared to the reference ligands. A distance is measured between the properties of each input ligand and the properties of the reference ligands. The ligands that have the smallest distance are considered the most similar. When there are two or more reference ligands, the distance is measured as the distance to the nearest reference. The distance is computed as a Euclidean distance.

#### 2.2.4 Biochemical evaluation of the hit molecules

Shanghai Medicilon Biomedical Co., Ltd (https://www.medicilon.com.cn/) is a professional preclinical comprehensive research and development service CRO with a history of 19 years, providing comprehensive one-stop new drug research and development services that meet domestic and international application standards for pharmaceutical enterprises and research institutions worldwide. It is listed on the Shanghai Stock Exchange Science and Technology Innovation Board with the stock code of 6882021. We entrusted Shanghai Mediciloniomedical Co., Ltd. to conduct *in vitro* assay for hit molecules using the methods provided by the CD47/SIRPα binding kit (https://www.cisbio.cn/human-cd47-sirp-alpha-biochemical-binding-kit-44631).

The HTRF CD47/SIRPα binding assay was designed to measure the interaction between CD47 and SIRP alpha. Utilizing HTRF (homogeneous time-resolved fluorescence) technology, the assay enables simple and rapid characterization of compound and antibody blockers in a high throughput format. The interaction between CD47 and SIRP alpha was detected by using anti-Tag1 labelled with europium (HTRF donor) and anti-Tag2 labelled with XL665 (HTRF acceptor). When the donor and acceptor antibodies are brought into close proximity due to CD47 and SIRP alpha binding, excitation of the donor antibody triggers fluorescence resonance energy transfer (FRET) towards the acceptor antibody, which in turn emits specifically at 665 nm. This specific signal is directly proportional to the extent of CD47/SIRP alpha interaction. Thus, compounds or antibodies blocking the CD47/SIRP alpha interaction will cause a reduction in the HTRF signal.

We consulted the database and related literature to determine the preliminary screening concentrations. The maximum IC_50_ value of the active CD47 small molecular inhibitor is 50 μM, and the maximum measured concentration is 100 µM. Therefore, we set the two preliminary screening concentrations, which were 10 and 100 μM. The *in vitro* assay of the hit molecules was evaluated under the protocol of the CD47/SIRP alpha binding kits (https://www.cisbio.cn/human-cd47-sirp-alpha-biochemical-binding-kit44631). For every concentration point of every molecule, a repeated point was conducted.

The ratio was calculated according to the following equation: emission ratio (ER) = Em665/Em615. Then, the ER of the compound was recorded as ER compound, the ER of the vehicle control was recorded as ER vehicle, and the ER of the blank control was recorded as ER blank. The inhibition rates at the two concentration points (10 μM and 100 μM) were calculated to indicate their ability to inhibit CD47-SIRPα binding. The inhibition rate was calculated by the following formula:

$$\text{Inhibition Rate}=\left(\text{ER vehicle }–\text{ ER compound}\right)/\left(\text{ER vehicle}-\text{ER blank}\right)\times 100\%$$

## 3 Results and discussion

### 3.1 Performance of gcforest with less training data

The architecture and training process of gcforest for CDK4 drug screening can refer to the architecture and training process of gcforest for face recognition task (Zhou and Feng, 2017). We adjusted the dimensions of multi-grand scanning windows, specifically, the original input dimension of the raw feature vectors of images of drug structures is 28*28, and 3 window sizes are used for multi-grained scanning, 7*7, 10*10 and 13*13 respectively. We use accuracy (ACC) and the screened active drugs to compare the performace of gcforest with CNN based CDK4 drug screening model (Xu et al., 2018). The results showed that the drugs predicted by gcforest include indocyanine green, and the accuracy was 91.35% (the most active CDK4 inhibitor predicted by CNN was indocyanine green, and the accuracy was 91.92%). We deleted the rotated compound images in the training set while gcforst could still screen out indocyanine green, and the accuracy was 89.43%. These results can fully prove the competence of gcforest in the case of less training data.

### 3.2 Performances on the benchmark datasets

**FIGURE 5:**
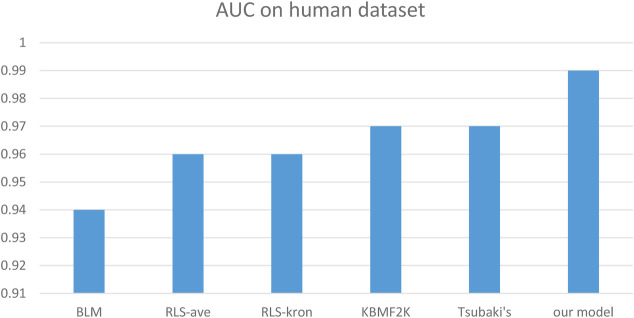
Performances of our proposed model and the specialized CPI prediction models and Tsubaki’s model on Human Dataset.

**FIGURE 6:**
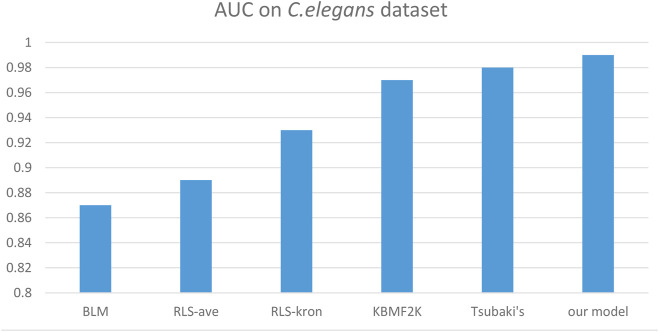
Performances of our proposed model and the specialized CPI prediction models and Tsubaki’s model on *C. elegans* dataset.

We compared the performance of the proposed model with other CPI prediction models on the human and *C. elegans* datasets constructed above. The evaluation metrics are AUC, precision and recall. The performances of all the CPI prediction models except our proposed model are obtained from the literature (Tsubaki et al., 2019). The ML based CPI prediction models’ environments in the literature are as follows: k-NN and RF were run by Weka 3.7, L2 was run by Liblinear 1.94, and the SVM was run by libsvm 3.17 (Tsubaki et al., 2019). Our model was run in the Ubuntu system and python 3.7 environment. And other ML CPI prediction models used the manual extracted features, such as the PubChem fingerprint and Pfam domain (Tsubaki et al., 2019). As shown in Tables 3, 4, on the human dataset, our model achieved significantly better performance compared with other models: k-NN, random forest (RF), logistic-2 (L2), SVM and Tsubaki’s model, while the precision and recall were only slightly less than that of the SVM. On the *C. elegans* dataset, our model is significantly superior to other methods on all evaluation metrics. We also compared the proposed method with other existing methods specifically for CPI prediction, i.e., BLM (Bleakley and Yamanishi, 2009), RLS-avg and RLS-Kron classifiers with GIP kernel (Laarhoven et al., 2011), KBMF2K classifier and KBMF2K regression (Gonen, 2012), which were running on the same experimental settings as Liu’s (Liu et al., 2015). Figures 5, 6 show the AUC scores on the human and *C. elegans* datasets. As can be seen, on both humans and *C. elegans* datasets, our model is superior all other methods. The above results fully demonstrated that the proposed method based on multi-grained cascade forest classifier and word2vec embedding tool to construct the model from raw data has great advantages compared with other CPI prediction models.

**TABLE 3:** Performances of our proposed model and other ML models on human dataset.

Metrics	k-NN	RF	L2	SVM	Tsubaki’s	Our proposed model
AUC	0.860	0.940	0.911	0.910	0.970	0.990
Precision	0.798	0.861	0.891	0.966	0.923	0.965
Recall	0.927	0.897	0.913	0.950	0.918	0.932

**TABLE 4:** Performances of our proposed model and other ML models on *C.elegans* dataset.

Metrics	k-NN	RF	L2	SVM	Tsubaki’s	Our proposed model
AUC	0.858	0.902	0.892	0.894	0.978	0.994
Precision	0.801	0.821	0.890	0.785	0.938	0.960
Recall	0.827	0.844	0.877	0.818	0.929	0.962

**FIGURE 7:**
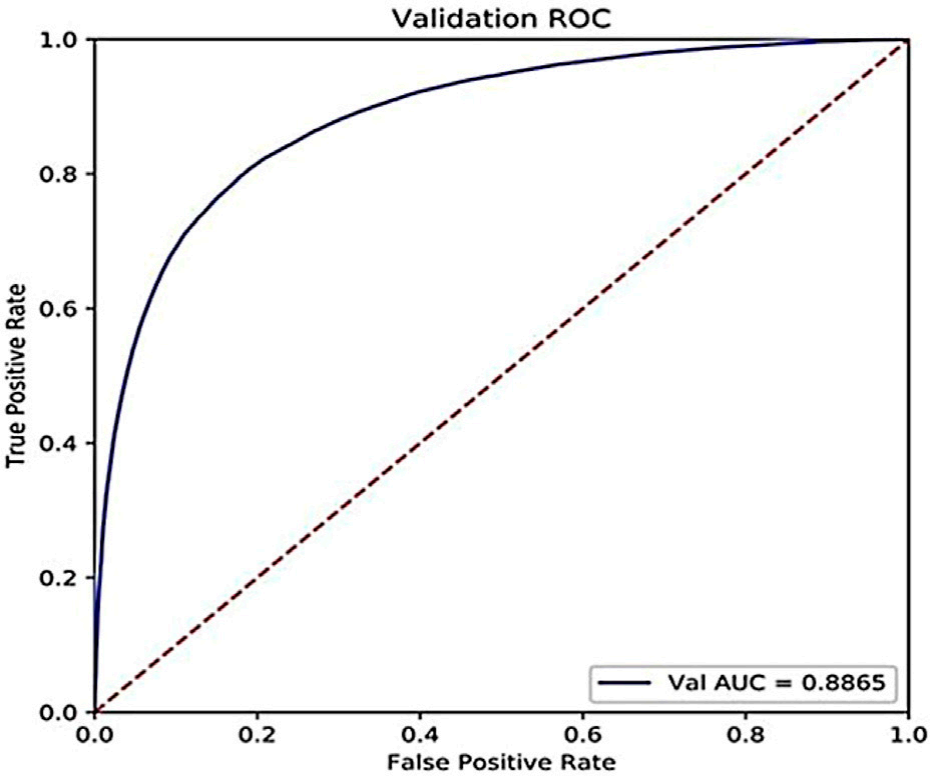
ROC curve of our proposed model on the constructed challenging dataset.

In addition, the accuracy on the challenging dataset constructed above is 85.21%, and the AUC is 0.8865, as shown in Figure 7. We can see that our model can still perform well on the challenging dataset where the percentage of the training set is only 20% to mimic the real scene that the number of the known drugs for a specific target is small.

Our proposed model achieved satisfying performances on both training and test datasets of the constructed latest bindingDB dataset. Similar to the performance of the recent popular end-to-end learning frame DeepLPI (Wei et al., 2022), which achieved AUC of 0.95 and 0.89 respectively for the training and validation on the bindingDB dataset, the performance on the validation set is not perfect but real enough, DeepLPI (Wei et al., 2022) also used SMILES of compounds and amino acid sequences of proteins as input. The performances of our model on the latest BindingDB dataset are summarized in Table 5. These results fully demonstrate the effectiveness and robustness of our model.

**TABLE 5:** Performances of our proposed model on the constructed latest BidningDB benchmark dataset.

	Accuracy (%)	AUC
Training set	95.50	0.9629
Test set	79.71	0.8685

### 3.3 Application to screen new CD47 inhibitors

We used the proposed models trained with 2 different datasets mentioned above to screen the commercial library. The inputs of the two screening models are the low-dimensional vectors of SMILES of molecules of specs and amino acid sequences of CD47 generated by word2vec, which are in the same form as the inputs of the corresponding training models. The only difference is that the model trained with the whole challenging dataset takes both SMILES and amino acid sequences as inputs, while the model trained with the known CD47 inhibitors takes only SMILES as inputs. The outputs of the two screening models are the predicted probabilities of being positive inhibitors for each molecule in the commercial library. The higher the probability, the more likely the molecule is to inhibit CD47. We selected 30 small-molecule compounds by applying a probability threshold of 0.515 to both models and choosing the molecules that met this criterion in both models. This means that the selected molecules have a high probability of being positive inhibitors for CD47 according to both models. A higher threshold would result in fewer hits, but a higher confidence, while a lower threshold would result in more hits, but a lower confidence. By choosing a threshold of 0.515, the screening model aimed to balance these two factors and select an appropriate amount of hit molecules for further validation.

The information of the 30 hit small molecules and the preliminary screening assay results are shown in Table 6. SWY-AK-309 and SWY-AM-598 showed a strong ability to inhibit CD47/SIRP alpha binding with activity less than 10 μM.

**TABLE 6:** Information of the 30 hit small molecules and the preliminary screening results.

ID	Structure	Purity	Mol weight	Preliminary activity
SWY-AF-060	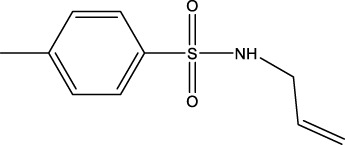	>95%	211.28	>100 μM
SWY-AG-052	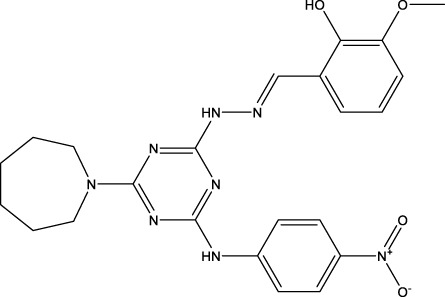	90%	478.51	>100 μM
SWY-AG-115	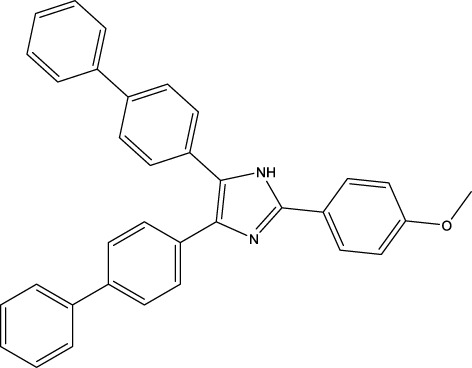	90%	478.59	>100 μM
SWY-AG-752	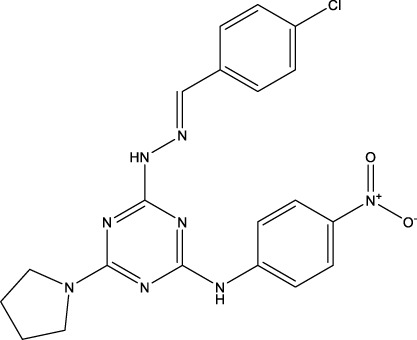	90%	438.88	>100 μM
SWY-AG-194	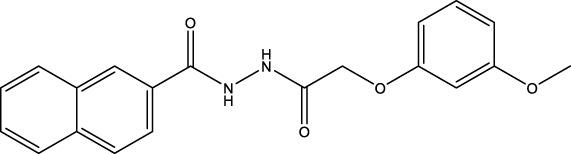	>90%	350.37	>100 μM
SWY-AG-025	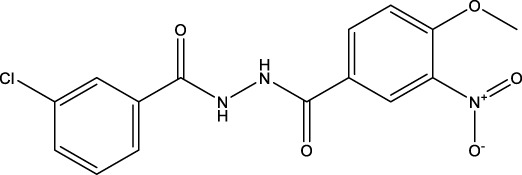	95%	349.73	>100 μM
SWY-AG-217	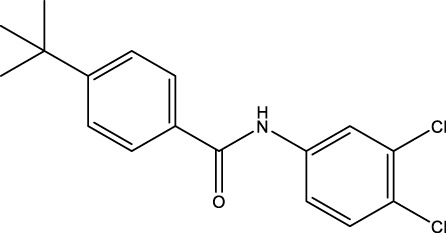	95%	322.23	>100 μM
SWY-AG-020	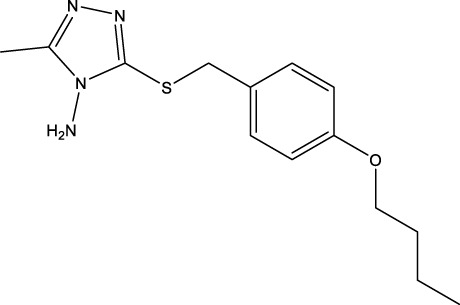	>95%	292.41	>100 μM
SWY-AG-101	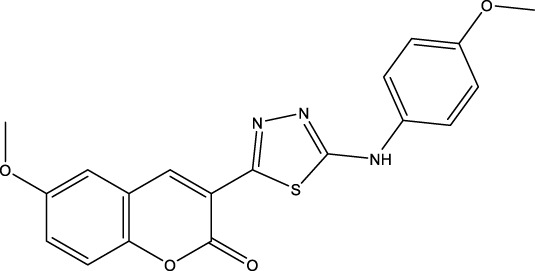	>95%	381.41	>100 μM
SWY-AG-660	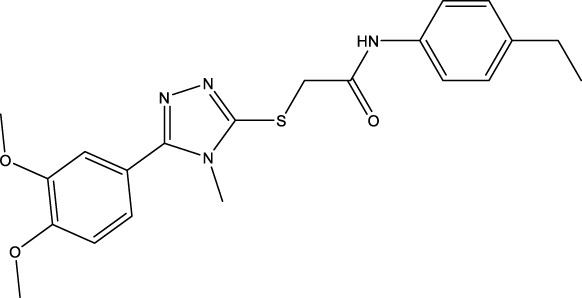	>95%	412.51	>100 μM
SWY-AG-490	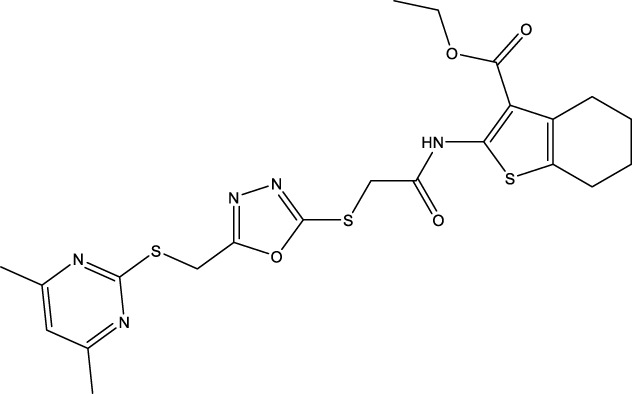	90%	519.67	>100 μM
SWY-AH-010	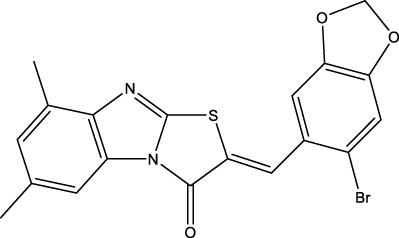	95%	429.29	>100 μM
SWY-AI-116	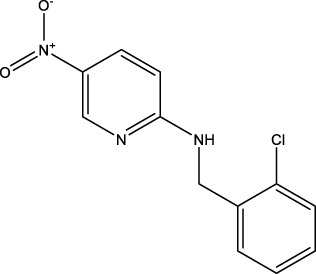	>95%	263.68	>100 μM
SWY-AF-282	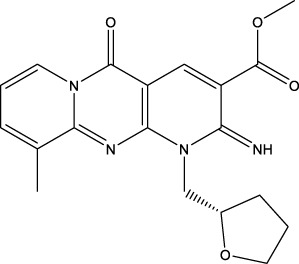	95%	368.39	>100 μM
SWY-AJ-008	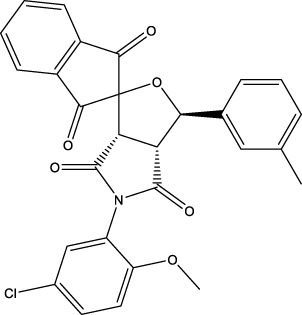	90%	501.92	>100 μM
SWY-AK-309	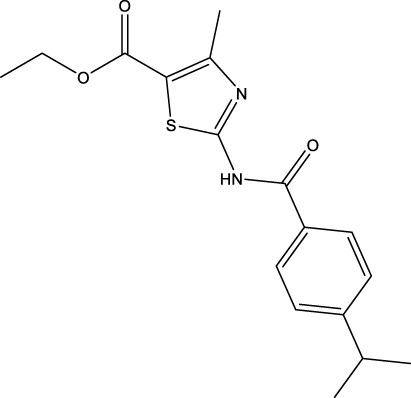	>95%	332.42	**<10 μM**
SWY-AK-653	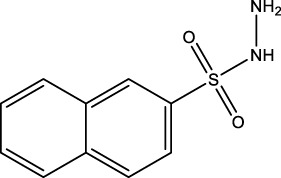	90%	222.27	>100 μM
SWY-AK-624	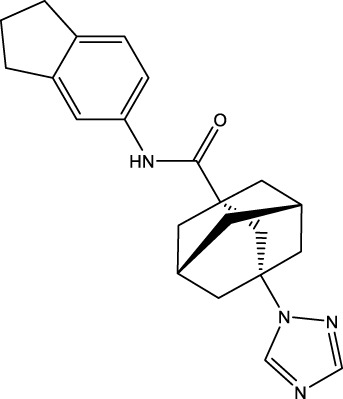	95%	362.47	>100 μM
SWY-AK-850	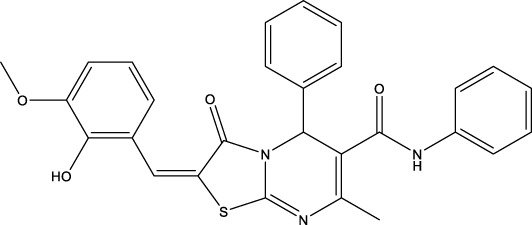	90%	497.57	>100 μM
SWY-AK-691	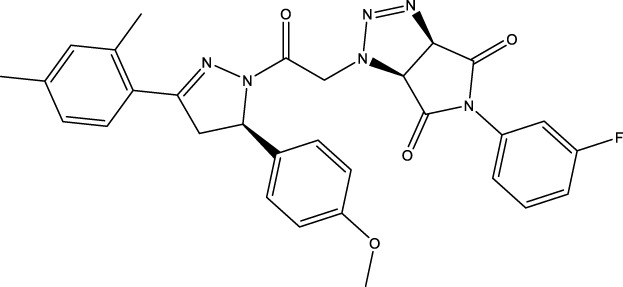	95%	554.58	>100 μM
SWY-AM-335	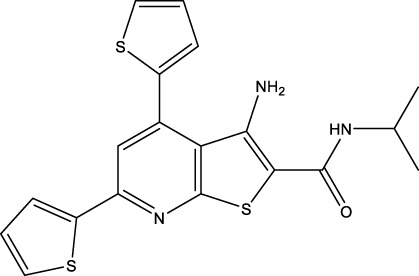	>95%	399.56	>100 μM
SWY-AM-262	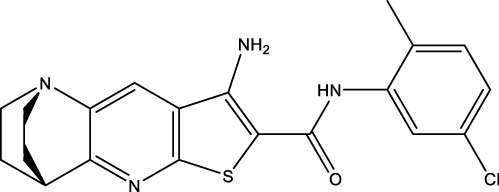	>95%	398.92	>100 μM
SWY-AM-009	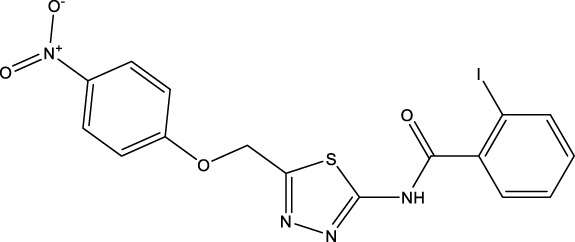	>95%	482.25	>100 μM
SWY-AM-598	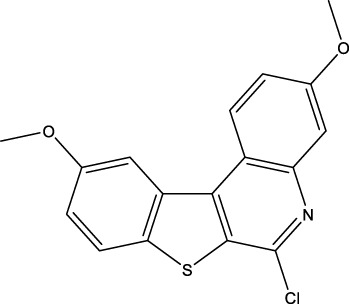	>95%	329.81	**<10 μM**
SWY-AN-658	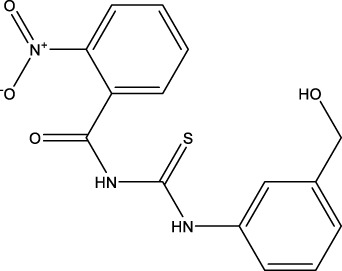	>90%	331.35	>100 μM
SWY-AN-823	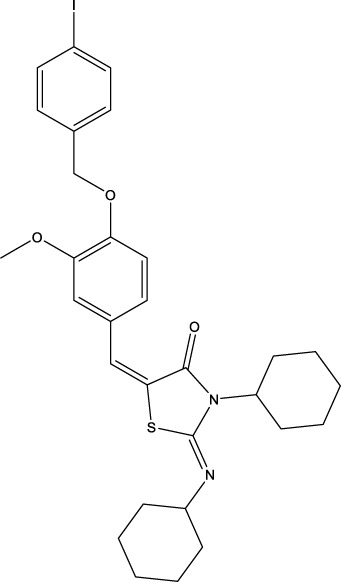	95%	191.27	>100 μM
SWY-AN-001	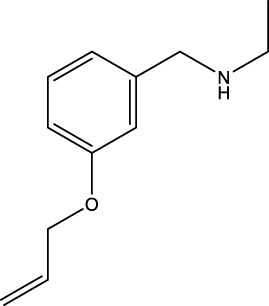	90%	630.58	>100 μM
SWY-AO-102	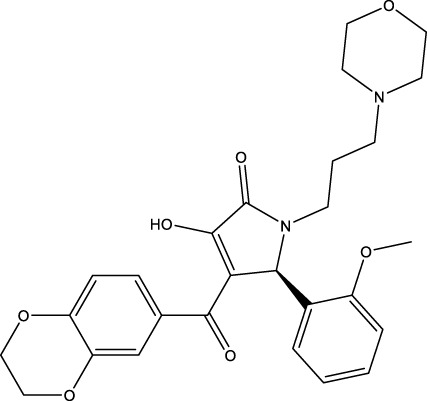	>95%	494.54	>100 μM
SWY-AO-756	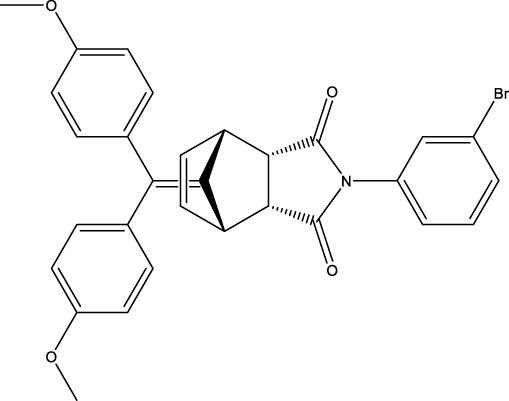	90%	542.43	>100 μM
SWY-AP-110	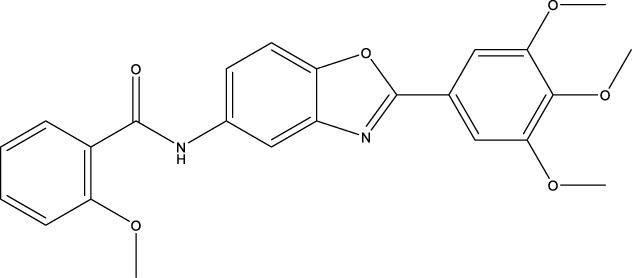	>95%	434.45	>100 μM

**FIGURE 8:**
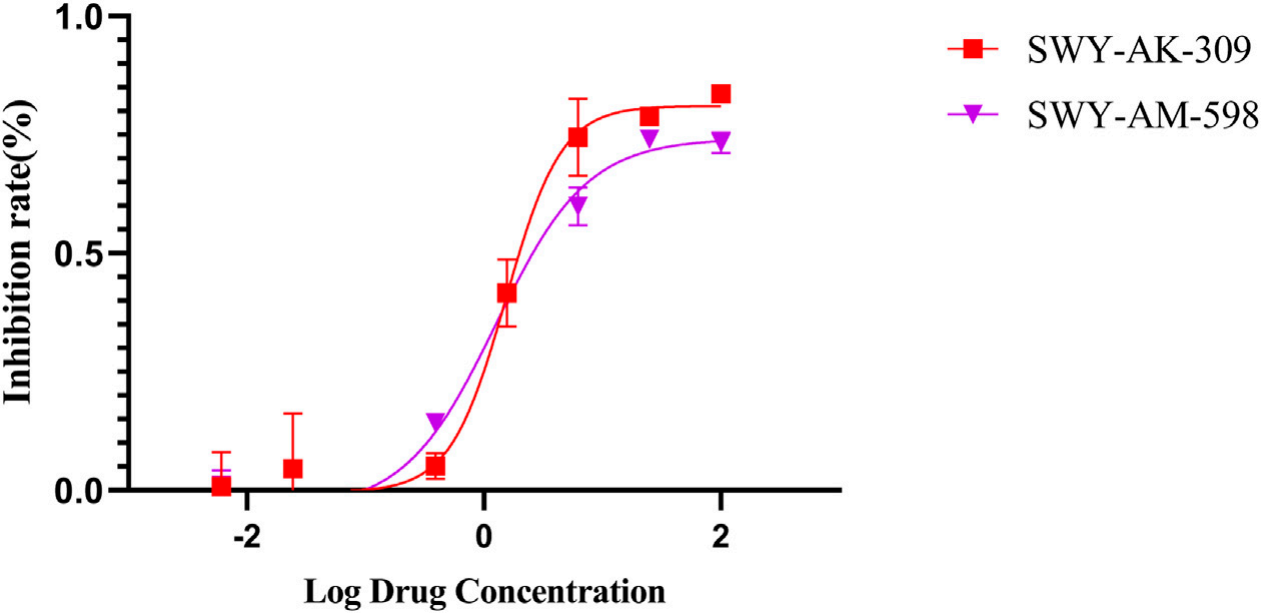
IC_50_s of the 2 most active molecules in the preliminary screening assay.

The IC_50_ values of the 2 most active molecules in the preliminary screening assay are shown in Figure 8, which are SWY-AK-309 and SWY-AM-598 with IC_50_s of 3.57 and 4.79 μM, respectively. These results fully demonstrate the efficiency of our proposed CPI prediction model.

**FIGURE 9:**
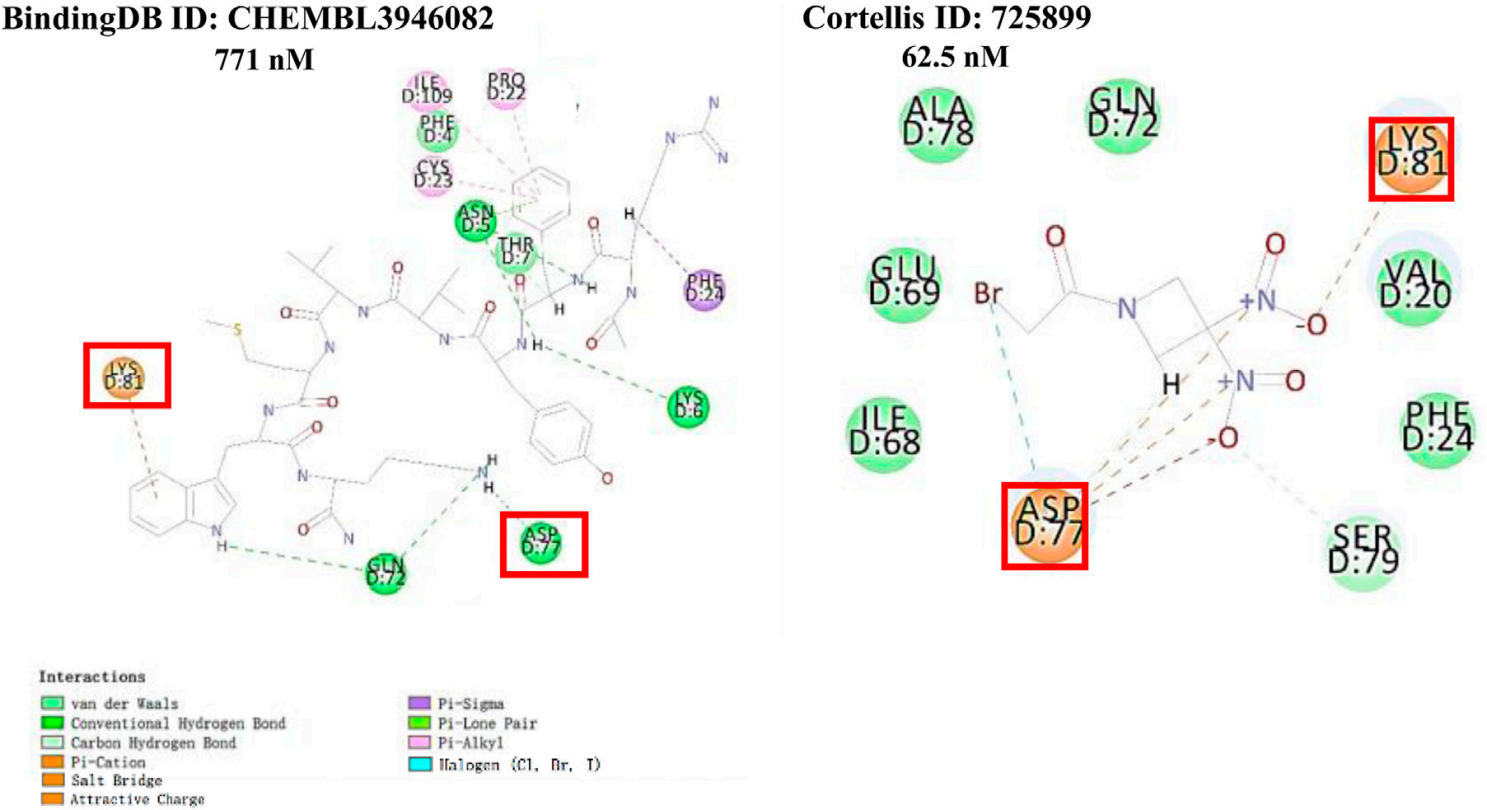
Interactions of the 2 known most active small molecular inhibitors with CD47 binding pocket.

**FIGURE 10:**
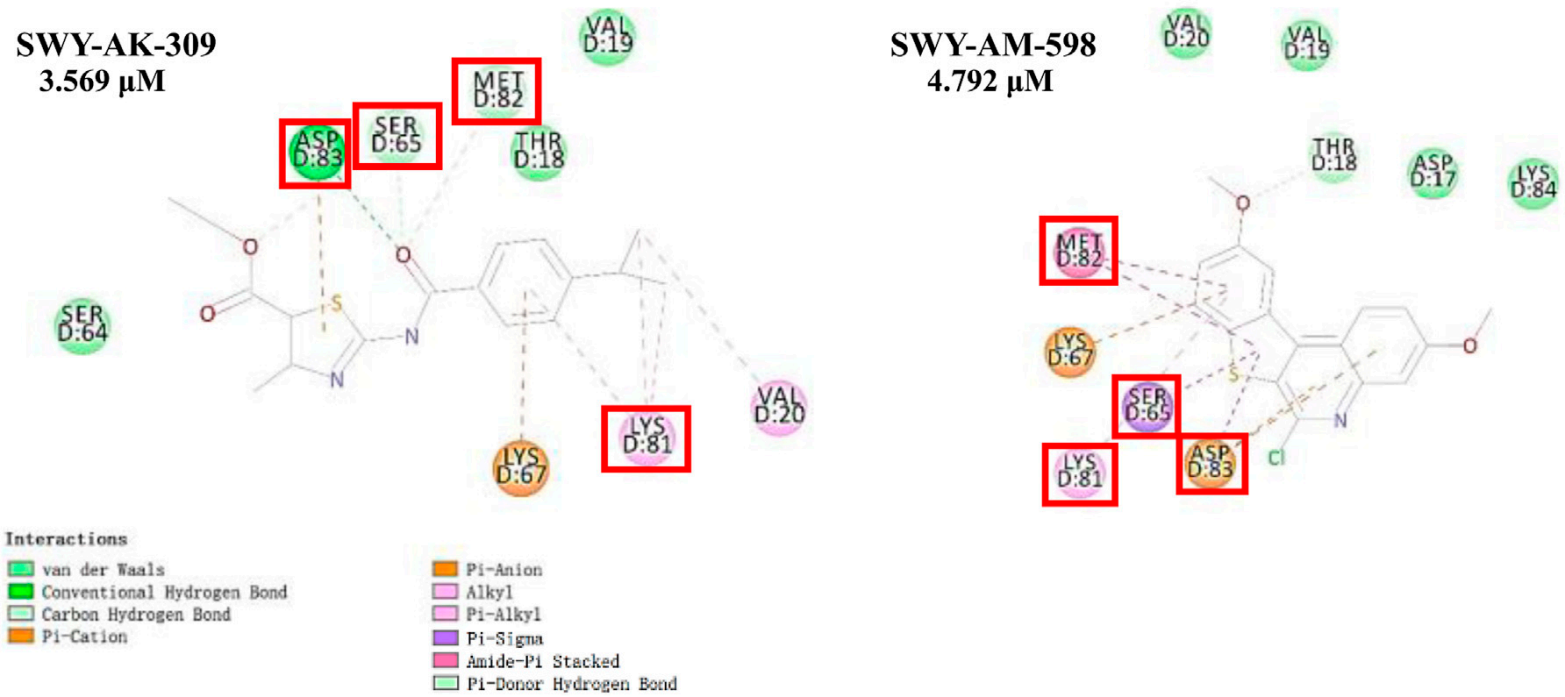
Interactions of the predicted 2 active inhibitors with CD47 binding pocket.

**FIGURE 11:**
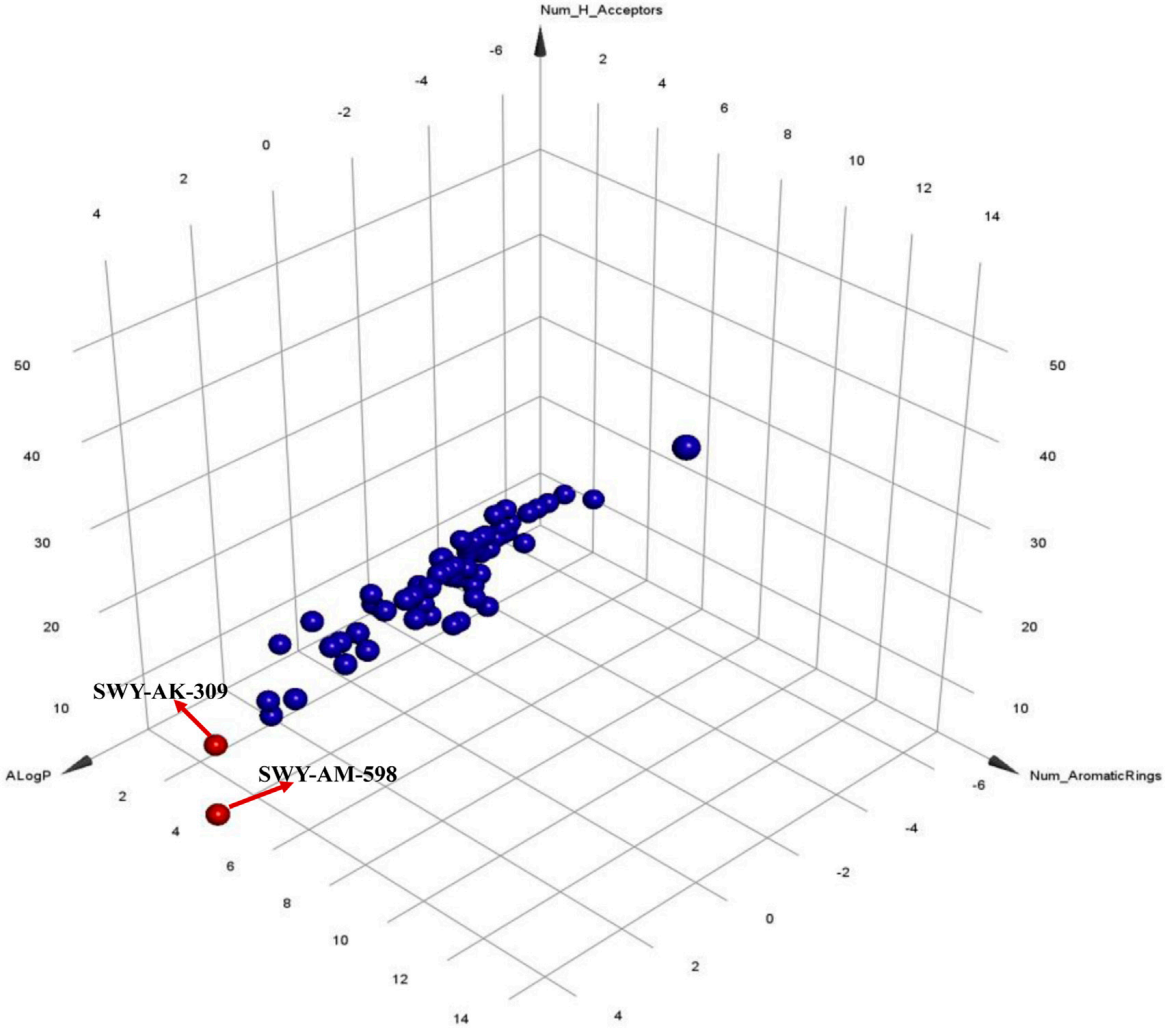
Visualization of the similarities between the screened inhibitors and the known inhibitors.

The 2 small molecules with the highest known CD47 inhibitory activity do not have much structural similarity, but the screened 2 active inhibitors are similar to one of the known active inhibitors, both containing aromatic rings and amide segments. We used docking to visualize the compound-protein interactions and find similarities between the known active inhibitors and the predicted inhibitors. We found that the 2 known most active CD47 inhibitors in Table 2 all interact with the binding pocket with two key residues in CD47, which are LYS81 and ASP77, respectively, as shown in Figure 9. We also docked the predicted 2 active molecules into CD47 pocket, as shown in Figure 10, SWY-AK-309 and SWY-AM-598 show similar interactions, in addition to the key residue LYS81 mentioned above in the analysis of the 2 known most active inhibitor, they all interact with residues like SER65, MET82, ASP83, which indicates the importance of these residues and may be useful clues for future molecule design. As shown in Figure 11 to visualize the similarities between the screened two active CD47 inhibitors with the 68 known inhibitors. The red points are the screened inhibitors and the blue point are the known inhibitors, the *X*-axis represents the ALogP, the *Y*-axis represents the Num_AromaticRings and the *Z*-axis represents the Num_H_Acceptors. We can see that the red points representing SWY-AK-309 and SWY-AM-598 are all close to the known inhibitors in the 3D space formed by the above 3 different axis properties, and SWY-AK-309 is closer with the known inhibitors than SWY-AM-598, which may explain its better activity. These results suggest that our models have learned some important features of CD47 inhibition and can screen new inhibitors that have similar properties. We also used SwissADME (Daina et al., 2017) to evaluate the drug-likeness of the predicted two new inhibitors and we find that the Lipinski, Ghose, Veber, Egan features all passed the standards without violation.

## 4 Conclusion

In this research, we suggested a unique CPI prediction model that benefits of end-to-end learning and ensemble learning. We utilized word2vec to generate low-dimensional vectors of SMILES of drugs and amino acid sequences of targets and a multi-grained cascade forest as the classifier to predict CPIs, enabling the model construction from raw data. Furthermore, our model can adaptively determine the complexity of the architecture according to the scale of dataset. Therefore, the model can perform well on small-scale datasets without many hyper-parameters and over-fitting compared with DL-based models. The suggested model outperformed the benchmark datasets, predicting two new small molecular inhibitors for CD47 which has few known inhibitors. We demonstrated that our suggested model is a succinct but efficient tool for CPI prediction through a series of optimization, validation and practical application in a specific target CD47. Our research group has applied this model to other targets and demonstrated good generalization ability (to be described in another article). Our paper focuses on the computational prediction and screening of CD47 inhibitors, which is a preliminary step in drug discovery. The toxicity of the discovered CD47 inhibitors in human cells is a complex and important issue that requires further experimental validation and evaluation.

In a word, our proposed model has few hyper-parameters and is competent on any scale datasets without over-fitting, especially for the specific target with few known drugs. Therefore, we believe that our model can overcome some of CPI’s challenges, serve as a concise but efficient tool to facilitate virtual screening, and be greatly efficient in more drug discovery scenarios.

## Data Availability

The datasets presented in this study can be found here: https://github.com/SylviaSWY/GCVec/.

## Abbreviations

CD47, Cluster of differentiation 47; AI, Artificial intelligence; ML, Machine learning; DL, Deep learning; CNN, Convolutional Neural Network; SMILES, Simplified molecular input line entry specification; GcForest, Multi-grand cascade forest; SIRPa, Signal regulated protein alpha; ACC, Accuracy; AUC, Area under ROC curve; SE, Sensitivity; SP, Specificity; FRET, Fluorescence resonance energy transfer; SVM, Support vector machine; RF, Random forest; L2, L2 regression.
